# Plant Diversity Conservation Challenges and Prospects—The Perspective of Botanic Gardens and the Millennium Seed Bank

**DOI:** 10.3390/plants10112371

**Published:** 2021-11-03

**Authors:** Elinor Breman, Daniel Ballesteros, Elena Castillo-Lorenzo, Christopher Cockel, John Dickie, Aisyah Faruk, Katherine O’Donnell, Catherine A. Offord, Samuel Pironon, Suzanne Sharrock, Tiziana Ulian

**Affiliations:** 1 Royal Botanic Gardens, Kew, Wakehurst, Ardingly, Haywards Heath, West Sussex RH17 6TN, UK; D.Ballesteros@kew.org (D.B.); E.CastilloLorenzo@kew.org (E.C.-L.); C.Cockel@kew.org (C.C.); j.dickie@kew.org (J.D.); A.Faruk@kew.org (A.F.); t.ulian@kew.org (T.U.); 2 Botanic Gardens Conservation International, Descanso House, 199 Kew Road, London TW9 3BW, UKSuzanne.Sharrock@bgci.org (S.S.); 3 The Australian Plant Bank, Australian Institute of Botanical Science, Australian Botanic Garden, Mount Annan, Sydney, NSW 2567, Australia; Cathy.Offord@botanicgardens.nsw.gov.au; 4 Royal Botanic Gardens, Kew, Kew Green, Richmond, Surrey TW9 3AE, UK; S.Pironon@kew.org

**Keywords:** biodiversity, long-term conservation, plant populations, strategic collecting, exceptional species, collection quality, safety duplication, seed longevity, seed viability, viability monitoring, integrated conservation

## Abstract

There is a pressing need to conserve plant diversity to prevent extinctions and to enable sustainable use of plant material by current and future generations. Here, we review the contribution that living collections and seed banks based in botanic gardens around the world make to wild plant conservation and to tackling global challenges. We focus in particular on the work of Botanic Gardens Conservation International and the Millennium Seed Bank of the Royal Botanic Gardens, Kew, with its associated global Partnership. The advantages and limitations of conservation of plant diversity as both living material and seed collections are reviewed, and the need for additional research and conservation measures, such as cryopreservation, to enable the long-term conservation of ‘exceptional species’ is discussed. We highlight the importance of networks and sharing access to data and plant material. The skill sets found within botanic gardens and seed banks complement each other and enable the development of integrated conservation (linking in situ and ex situ efforts). Using a number of case studies we demonstrate how botanic gardens and seed banks support integrated conservation and research for agriculture and food security, restoration and reforestation, as well as supporting local livelihoods.

## 1. Introduction

We are living in a time of unprecedented change. In the past 50 years, the human population has doubled, while the global economy has quadrupled, and global trade has increased ten-fold [1]. This has resulted in a concomitant increase in demand for resources and energy, the consumption of which is having profound impacts on the natural world. Human economic activities are driving climate change and biodiversity loss, both of which are mutually reinforcing, further exacerbating the problem [2,3]. In 2020, for the first time, environmental issues dominated the top five global risks by likelihood, as identified by the World Economic Forum, with biodiversity loss, climate action failure and extreme weather also all in the top five risks with greatest impact [4].

Human actions have significantly altered 75% of the land surface of our planet and led to an increase in the rate of biodiversity loss unparalleled in human history [1]. In relation to plants, this has resulted in a ~50% reduction in plant biomass relative to pre-human levels [5], and to ~40% of plant species being threatened with extinction [6]. Conservation interventions are urgently required to help reverse these trends and ensure that adequate plant diversity is available for both current and future generations. All life depends on plants. We rely on them, and the fungi that support them, for our foods, materials, medicines, and for regulating, supporting and cultural ecosystem services. The Dasgupta Review [7] demonstrates how our economies, livelihoods and wellbeing are dependent on nature, and embedded within nature, and calls for transformative change to put the sustainable use of nature at the heart of our economies.

In the face of a sixth mass extinction [8], the conservation of plant diversity has never been more important for the future of the planet and people. Protecting plants in their natural environment (i.e., in situ conservation) is the primary approach for species conservation, but as many of the threats to their continued existence (e.g., climate and land use change, invasive species, pollution) do not respect the boundaries of protected areas a safety back up is required. Conserving plants away from these threats (i.e., ex situ conservation), in botanic gardens (as living collections of plants) and seed banks (as propagules), is crucial if we are to stop and even reverse the extinction trend and preserve plant diversity for current use and for future generations. Storing representative germplasm from plants (any part of the plant that can be used to regenerate a new individual) collected from the wild ex situ facilitates the utilisation of this material for a variety of purposes without impacting the wild population. So not only does it protect the plant material from the threats faced in situ, it provides a readily accessible resource for use.

The conservation of living plant material in these human-made repositories has been practiced for centuries, but in recent decades has greatly expanded and become increasingly refined. To date, more than 105,600 wild plant species are conserved as seed and/or living collections in botanic gardens [9]. Their conservation takes the form not only of the living plant, seed or tissue, but also encompasses the knowledge and data associated with the individual samples and collections, and the ability to communicate this to a wide variety of audiences to improve conservation outcomes.

**Figure 1:**
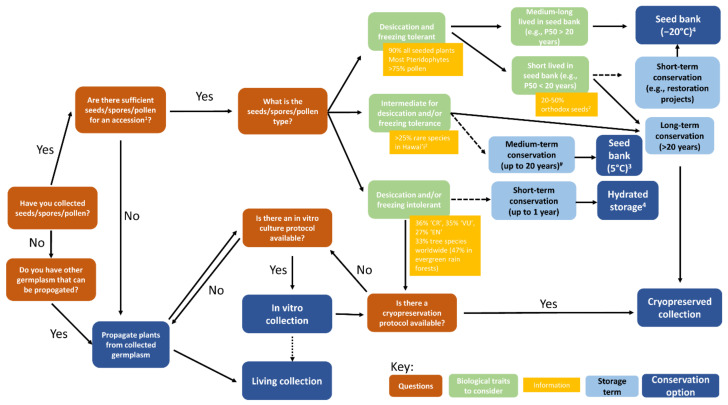
Decision tree showing conservation options depending on the type of germplasm conserved and some of the biological traits associated with different germplasm. Short- and medium-term storage options are indicated by dashed arrows. In vitro collections can be a source of germplasm for living collections (dotted arrow). CR: critically endangered species, EN: endangered species, VU: vulnerable species as per IUCN Red List. # refers only to desiccation tolerant but freezing sensitive seeds. ^1^ [14,15,16,17], ^2^ [12,18], ^3^ [19], ^4^ [20].

Botanic gardens have a long history of conserving plant diversity through their living collections. These are generally raised from material collected in the wild (Figure 1), and often include threatened species. However, the primary method of ex situ conservation for plant genetic resources today is seed banking (Figure 1). While the function and name of the seed bank (genebank, germplasm bank, biobank) may vary, the general concept remains the same—using controlled environments (drying and cooling) to preserve a broad diversity of plant germplasm for immediate and future use. This methodology underpins global food security, forestry, horticulture and ecological restoration as well as conservation. While the scale of the seed bank, the size and diversity of the seed accessions held, the post-harvest handling and storage procedures employed, and the extent of environmental controls used may vary, the purpose remains the same—to preserve high-quality, viable germplasm until required for use [10]. 

However, a wide variety of plant diversity, referred to as ‘exceptional species’ [11,12], cannot be stored for the long-term under conventional seed banking methods (commonly drying to 15% relative humidity at 15 °C, followed by storage at −20 °C in airtight containers). Examples include gametophytes of non-seed-bearing plants (e.g., bryophytes and pteridophytes), germplasm with desiccation and/or freezing intolerance (e.g., *Quercus* seeds), and species with desiccation and freezing tolerant germplasm (both spores and seeds) that is short-lived under conventional seed bank conditions. Such ‘exceptional species’ can form a large part of the global flora; for example, it is estimated that at least 8% of all flora, and 18% of tropical and sub-tropical flora, are likely to have desiccation sensitive (recalcitrant) seeds [13]. Other options for the conservation of such species include tissue culture, cryopreservation, nursery-based plant collections and seed orchards (Figure 1). 

Botanic Gardens Conservation International (BGCI) was established in 1987 with a particular focus on identifying threatened species in botanic garden collections and supporting ex situ conservation efforts for these species. There are presently over 3000 botanic gardens located in 185 countries included in BGCI’s GardenSearch database [21], many of which are actively engaged in conservation action for threatened plant species. Recently, efforts have been focused on establishing consortia of gardens with common conservation interests to establish decentralised metacollections. The increasing use of metacollections across botanic gardens and arboreta has led to a step change in the importance of these collections for plant conservation [22,23]. 

The Millennium Seed Bank (MSB) of the Royal Botanic Gardens, Kew (Kew), was established in 2000 building on more than three decades of research at Kew into seed biology and conservation. The MSB’s mission is safeguarding wild plant diversity and enabling its sustainable utilisation through global partnership. To this end, the Millennium Seed Bank Partnership (MSBP), consisting of seed banks associated with botanic gardens, agricultural, forestry or research institutes, and government organisations around the world, has been collaboratively conserving native floras. To date, more than 97 countries and territories and over 250 organisations have been involved. When referring to seed material collected of wild origin stored within seed banks for long-term conservation, we use the term ‘accession’. Each accession represents material collected from an individual population (unless maternal lines are stored individually) at a given time, and multiple accessions of an individual species may be held. 

With the increased focus on plant conservation provided by the Global Strategy for Plant Conservation (GSPC, 2011–2020), notably Target 8 [24], and the continued call for plant conservation to support the UN Sustainable Development Goals [25], the number of botanic gardens working in conservation, and the number of seed banks conserving wild species has greatly increased. However, there is a disparity between the location of biodiverse, threatened habitats and the sites of these conservation centres [26]. The policy framework provided by the Convention of Biodiversity and its Nagoya Protocol [27] is crucial for ensuring that plant germplasm collected from the wild and stored, often in remote locations, is managed and utilised to ensure equitable benefit-sharing.

In this review, we focus on the challenges and prospects facing the long-term conservation of plant diversity in botanic gardens’ living collections and seed banks. Having evaluated the unique role that both forms of conservation play, we highlight the synergies afforded when they are co-located in the same botanic garden and the importance of networks for enabling plant conservation. We provide examples of how wild plant collections in both botanic garden living collections and seed banks support plant conservation in situ in the context of restoration, agriculture, forestry and livelihoods, with a focus on the challenges and prospects of these programmes in relation to long-term plant conservation ex situ. We finish by signposting three key areas for future development: funding security for ex situ collections; exploiting technological advances, specifically to enable the conservation of ‘exceptional species’; and maintaining and developing networks to ensure the best outcomes for the world’s flora.

## 2. Ex Situ Conservation

### 2.1. Botanic Gardens 

The practice of cultivating plants in specialised gardens has been around for thousands of years. However, the first ‘true’ botanic gardens with an underlying scientific foundation were the physic gardens of Italy created in the 16th century [28]. These gardens were purely for the study of the healing properties of medicinal plants and by the end of the century had spread to universities and apothecaries throughout Europe [28].

Botanic gardens experienced a change in usage during the 17th and 18th centuries. This was the age of exploration and the beginnings of international plant trade. Gardens such as Kew and the Real Jardín Botánico de Madrid were set up to try and cultivate ‘new’ species that were being brought back from expeditions to the tropics. Not only did these gardens promote and encourage botanical exploration, but they also helped found new gardens in the tropical regions to help cultivate these newly ‘discovered’ plant species, as well as providing a garden environment that ex-patriots recognised from home. During the 19th and 20th century, municipal and civic gardens were created around the world. However, many of these gardens were pleasure gardens with very few of them having any scientific programmes. Meanwhile, especially amongst university gardens and building on the work started by Linnaeus in the 18th century, collecting, naming and describing plant diversity became a major focus of activity and their diverse living and herbarium collections supported the teaching and practice of taxonomy. 

In the last 50 years, there has been an exceptional growth in the establishment of botanic gardens, with 60% of the gardens in existence today being established since the mid-20th century [29]. This growth has been particularly notable in China, where new gardens are being developed across the country with the aim of using local plant diversity to support economic development. Similarly, Indonesia plans to have a botanic garden in every province to function as a botanic resource centre to support conservation and sustainable development. Around the world, botanic gardens have seen a revival as scientific institutions due to the emergence of the conservation movement and the recognition of the importance, not only of their diverse collections, but also of the taxonomic and horticultural knowledge they possess, vital for the conservation, management and restoration of plant diversity. A botanic garden today can be defined as “an institution holding documented collections of living plants for the purposes of scientific research, conservation, display and education” [30].

BGCI underpins its work with three databases. The first, GardenSearch [21], is a directory of the world’s botanic gardens compiling information on their location and facilities, while PlantSearch [9] documents the plant collections held by these gardens. In order to identify threatened species in collections, BGCI has also developed a third database—ThreatSearch [31], which lists global, regional and national red list assessments for plants from a range of sources. It is the most comprehensive database of conservation assessments of plants. 

At the present time, GardenSearch includes details on 3715 botanical institutions, of which 3038 are botanic gardens (the remainder being a combination of seed banks (many of which are located within or connected to botanic gardens and maintain seed accessions of wild plant diversity (e.g., Kew’s MSB, which has its own listing), zoos and private collections). Over 350 of the botanic gardens listed in GardenSearch have established seed banks for the conservation of wild plant species [32]. PlantSearch presently contains 1,559,119 records, representing 634,235 taxa (species, varieties, sub-species and cultivars) held by 1186 institutions. An analysis of these records (excluding cultivars) carried out by Mounce et al. in 2017 [26], revealed that the global network of botanic gardens houses over 100,000 species of the 350,699 accepted plant species on The Plant List in 2013 [33]. These species are from nearly 10,000 genera, representing 30% of global plant species diversity, and over 41% of known threatened species. The botanic garden network is also rich in expertise, with more than 60,000 botanical specialists employed, covering plant sciences, taxonomy, education and specialist horticulture [34].

Most of the plant diversity held by botanic gardens exists in their living collections and not all of this diversity is being maintained for conservation purposes. Research, display, education and public outreach are also important functions of botanic gardens that are supported by the living collections. It is estimated that, globally, some 750 million people visit botanic gardens annually [34]. Therefore, as well as being important for conservation, the living collections of botanic gardens provide an important resource for educating and informing the public about the importance of conservation issues. Species that have memorable economic, ecological, or cultural stories are particularly useful in this regard, and help botanic gardens combat plant blindness [35].

As well as focusing on wild species, there are numerous examples where conservation by botanic gardens plays an important complementary role to that of the agricultural and forestry sectors [34]. For example, important collections of non-timber trees, such as fruit and nut species, exist in botanic gardens, and these might otherwise ‘fall through the cracks’ of plant conservation [36]. Examples include the breadfruit collection at the National Tropical Botanic Garden in Hawaii and the tropical fruit collection at Fairchild Tropical Botanic Garden in Florida.

#### Metacollections—Enhancing the Conservation Value of Living Collections

Some endangered plants are considered ‘exceptional species’ and do not store well in seed banks. Conservation efforts therefore rely heavily on living plant collections. Such collections need to include a high level of genetic diversity to ensure that the species can adapt and survive in the face of future changing environmental pressures. Curating a genetically diverse seed bank collection is relatively easy and affordable, but much more challenging for living plant collections. Balancing space and cost limitations while maximizing the number of individuals an institution can sustainably curate in its living plant collection is critical. The number of individual plants that are needed to capture a population’s genetic diversity varies considerably from species to species. This is illustrated by recent work on palms and cycads at the Montgomery Botanical Center [37]. For instance, for the rare Key Thatch Palm, *Leucothrinax morrisii*, curating 15 individuals can conserve as much as 83% of a population’s genetic diversity [38]. However, for the rare Sinkhole Cycad, *Zamia decumbens*, curating 30 individuals only conserves about 35% of the species’ genetic diversity. It may take more than 205 individuals to capture 77% of known genetic diversity for this cycad [39].

Given that any number of botanic gardens may hold collections of the same species, the conservation value of such collections can be considerably enhanced by combining these holdings into a network of collections—or a metacollection [40]. Metacollections are envisioned as common resources held by separate institutions but stewarded collaboratively for research and conservation purposes [22,23]. Networking multiple collections into a single metacollection increases potential coverage within a taxonomic group, allows broader access to greater diversity, dilutes risk of loss, and can reduce maintenance costs by reducing duplication and redundancy across collections. Like any collection, a metacollection can be of any scope or taxonomic level; however, the approach is particularly useful in the case of taxa that are only represented by a few individuals per garden—such as many tree taxa. The metacollection strategy was adopted by zoos over 40 years ago and is now embodied in the successful species management programmes for zoo animals. Gardens have been less formalised in networking plant genetic resources, but adapting zoo methods for plant collections is now yielding some important advances [41]. Established examples of botanical metacollections include the American Public Gardens Association’s Multisite Collections [42], BGCI’s Global Conservation Consortia [43] and the Center for Plant Conservation (CPC) National Collection [44].

As well as promoting the conservation value of living collections through the metacollection approach, in recent years BGCI has been active in supporting and encouraging the establishment of seed banks in botanic gardens as a complement to living collections to ensure the long-term conservation of native plant diversity. Such initiatives include the Global Seed Conservation Challenge (described below), training and capacity building activities and the provision of small grants for seed banking. The on-going development of an accession-level module as part of the PlantSearch database is also aimed at supporting a more cost-effective and coordinated approach to the conservation of threatened species across the botanic garden community.

### 2.2. Seed Banks 

In addition to the >350 wild plant seed banks established by botanic gardens [21], between 710 and 1750 genebanks exist, and maintain between 5.4 and 7.4 million accessions of plant genetic resources for food and agriculture, as seed, in vitro, DNA and cryopreserved collections [45,46]. Out of all these banks, two prominent examples are the Kew’s MSB (UK), and the Global Seed Vault (Svalbard, Norway); both exceptional in the extent of their international nature. The former is the world’s largest repository of wild plant genetic diversity, storing almost 997,000 accessions representing over 40,000 species, while the latter holds the world’s most diverse collection of food crop seeds with over 1.1 million seed accessions of around 4000 species. While many types of seed banks exist, for the purposes of this review we are focusing on those banking seeds collected from wild plant populations for long-term conservation, using the MSB and its global partnership, the MSBP, together with other seed banks in the BGCI network as our examples. 

#### 2.2.1. Botanic Garden Seed Banks

Of the botanic gardens listing seed banks as part of their facilities in GardenSearch, the majority are located in the global north, particularly in Europe and North America. Interest in using seed banks to conserve wild plants is relatively recent. Spain was one of the first countries to focus on collection of wild flora, creating its seed bank in 1966, at the Department of Plant Biology of the Polytechnical University in Madrid. Several Spanish botanic gardens then created seed banks focused on conserving wild flora in the regions in which they were based. Together, 10 of these seed banks formed the Spanish Network of genebanks for wild plants (Red Española de Bancos de Germoplasma de Plantas Silvestres) in 2002 [47]. 

**Figure 2:**
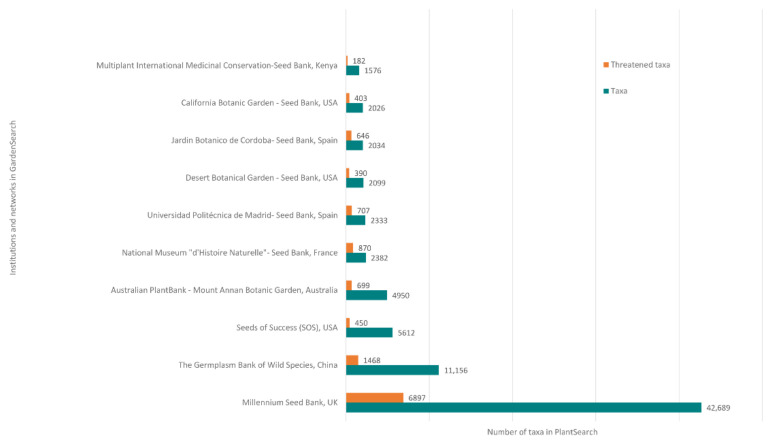
Seed collection holdings of the largest ten wild plant seed banks by the number of taxa stored based on PlantSearch data. Threatened taxa includes species categorised as threatened (including global, regional and national assessments) from data in ThreatSearch.

The number of seed banks in botanic gardens has doubled in the last 20 years and now the botanic garden community has some of the largest and most sophisticated seed banks in the world. As well as the MSB, such seed banks include the Germplasm Bank of Wild Species, located at the Kunming Institute of Botany in China which presently conserves over 11,000 species, and the Australian PlantBank that holds more than 12,000 seed accessions from almost 5300 plant species—437 of which are threatened species (G. Errington pers. comm.) (Figure 2). Of the records in PlantSearch, seed bank accessions represent 67,270 taxa in at least 100,218 accessions held in over 80 institutions. An analysis of seed bank data from PlantSearch and ThreatSearch [31] indicates that less than 10% of taxa in seed bank collections are globally threatened.

The skill sets developed in botanic gardens for the management of living collections and seed bank collections complement one another, and, together with the taxonomic skills of herbarium staff, provide the knowledge required for successful long-term ex situ conservation of plants. Excellent taxonomic and identification skills ensure that the correct plant material is sought and stored. Understanding of phenology and seed biology increases the success of fieldwork and storage. Propagation, both laboratory and nursery based, is vital for turning seeds back into plants that can be used. In addition, the education and outreach roles of botanic gardens enable the stories relating to the plants conserved, and the need for conservation itself, to be communicated to the public. It is not surprising, therefore, that the number of wild plant seed banks located in botanic gardens has seen a dramatic increase in recent years. 

Various manuals and guidelines have been created by the botanic garden seed bank community to ensure the quality of seed bank collections. An important aspect of the expansion of conservation efforts in Australia, is the progressive development of a suite of guidelines that capture the latest science and practice for seed banking and associated conservation activities—the Australian Germplasm Guidelines [48]. The publication is a collaboration of over 70 seed banks, botanic gardens and other organisations throughout Australia providing expertise in planning, collecting and management. The knowledge and skills shared in these guidelines are largely underpinned by contributions from Australian botanic gardens staff and their international collaborators. Of particular note in the latest edition is the expansion in information and techniques available for conservation of ‘exceptional species’ [12]. A major step forward is that requirements to conserve these species ex situ are being progressively understood and workflows have been developed to assist the fast-tracking of conservation efforts. This includes the latest techniques for selection of appropriate germplasm to conserve ex situ, an important step for the success of ex situ conservation of ‘exceptional species’ which require significant research effort [49]. At the regional level, the European Native Seed Conservation Network (ENSCONET), a consortium of organisations interested in native species conservation, created seed collecting and processing manuals for practitioners, available in nine European languages [50,51].

#### 2.2.2. The Millennium Seed Bank

The advantages of seed banking for the long-term preservation of wild plant diversity include the ability to store a wide range of genetic diversity in the form of seed accessions from populations, in a relatively small space (~40,000 species stored in 300 m^2^ at the MSB) and at a relatively low cost compared to other ex situ options (e.g., in vitro, field genebanks, etc.). Holding well documented collections also enables the current and future use of this germplasm resource. 

Kew has been working in seed banking since the 1970s, developing a seed banking programme for UK native species in the 1990s, and increasing its support of international plant conservation through seed banking since the 1990s. All the partners who have worked with the MSB since 2000, forming the MSBP, enter access and benefit sharing agreements with Kew to ensure equitable sharing of benefits and prior informed consent (PIC) relating to use of materials stored at MSB. They receive on-going support from the MSB in relation to development of seed banking facilities in their countries, training in seed conservation techniques, and in targeting and banking their most important parts of their floras. Many also undertake joint research programmes with Kew. The MSB works with a range of seed banking institutes, from those in botanic gardens (27%), to forestry and agricultural genebanks (20%), universities and other research organisations (32%), governmental (17%) and other organisations (4%). For example, the Royal Botanic Gardens and Domain Trust joined the MSBP in 2003 and, in 2013, opened the Australian Plantbank, a purpose-built conservation centre that incorporates the seed bank, alongside cryostorage, tissue culture and a well-developed nursery, with associated staff including a well-developed conservation focused science programme. 

The facilities at the MSB were built to last for 500 years, and seed accessions stored there should be viable for decades to centuries (depending on the species—some are short lived even under ideal storage conditions—and the quality of the accession). The long-term nature of this storage (>10 years) sets conservation seed banking apart from other seed storage initiatives with short (<5 years e.g., restoration and regeneration) and medium (5–10 years, e.g., plant breeding) storage needs, and defines the conditions of storage (Figure 1) [20].

The storage procedures and monitoring of long-term conservation collections of wild plant germplasm applied at the MSB, and botanic gardens seed banks generally, vary in some respects from those employed in other types of genebanks which follow the Food and Agriculture Organization of the United Nations (FAO) and the International Seed Testing Association (ISTA) guidelines [20,52], but are based on practice developed in this sector. Changes result from the diversity of wild seed types that are handled and stored (non-uniform seed material), and the long-term nature of the conservation collections (10–100 s of years). Storage conditions consist of drying to 15% equilibrium relative humidity and storing in glass containers or trilaminate foil bags at −20 °C. Protocols are adapted for short-lived species and for micro-seeds (<0.2 mm in length [53]). The MSBP Seed Conservation Standards [54] were developed to ensure high quality collections are made and stored across the partnership. The quality of an accession is assessed through the number of seeds in the accession together with their viability and longevity. The genetic representativeness of individual seed accessions and storing multiple accessions from different populations of the same species is also important. Seeds are stored in the country of origin with up to half of the collection sent to the MSB for duplicate storage, spreading risk by splitting the collections between two geographically separate facilities. Where no adequate facilities exist in country for the long-term storage of seed accessions, the whole collection can be sent to the MSB, and half returned on establishment of appropriate facilities in country. In some cases, for example, where legislation prevents the movement of national germplasm, seeds remain in the country of origin and are duplicated nationally, while data are shared with the MSB. Updates relating to the viability of accessions and requests for use of material are communicated to partners that store the duplicates. 

A conservation seed accession consists of three items: the seeds; an herbarium voucher from the population collected; the data associated with the accession (field, processing, germination, etc). The herbarium voucher enables identification of the seed material to be verified, and taxonomic changes to be tracked, while also providing a valuable ecological and historical record.

##### Collection Size

The quality of seed collected from wild plants varies significantly. A cut test to check seed quality prior to collecting is recommended. This enables an estimate of the number of potentially viable seeds in the population to be made, which will determine if sufficient high-quality seeds are available to enable a collection of 10,000 seeds to be made without impacting on the regeneration success of the wild population. The amount of seed collected should not exceed 10–20% of the seed available on the day of collection [55,56,57]. Collecting 10,000 seeds is recommended by the MSB to enable duplication between seed banks, routine seed bank activities and seed supply over the intended lifespan of the collection [14], it also allows for genetic attrition over time due to germination failure, disease and active use [58]. It is, however, recognised that collections of this size will not be possible for many rare and threatened species, and for those with restricted distributions, and a median of 1000 seeds per accession is more common and often still adequate for conservation purposes (Figure 1) [15,58]. For very rare plants, a seed collection of any size provides options for the future.

Accessions undergo X-ray analysis after cleaning and prior to banking to determine the proportion of full, potentially viable seeds within the sample. Many seed banks do not have access to an X-ray machine but can still undertake a cut-test to determine the quality of the seed. The number of seeds in an accession is identified either by direct count (for small accessions <500 seeds) or by weight (five samples of 50 seeds weighed, remainder of accession weighed, calculation performed). The original seed number is then adjusted using the X-ray (or cut-test) results to provide an estimate of the number of potentially viable seeds in the accession. Prior to sampling an accession, it is thoroughly mixed to ensure that seeds representative of the whole accession are utilised.

##### Genetic Representativeness

Conservation seed collections should contain genetic diversity that is representative of the population from which they were made, and collections should be made from enough populations to represent the genetic diversity of the species across its range. Multi-year collections from the same population may also be needed to capture the genetic diversity of annual or short-lived species. Typically, those making conservation collections aim to collect from at least 50 plants across the population, and to spread the number of seeds collected per plant evenly between individuals [59]. For large shrubs and trees, it is also recommended to collect from across the canopy [60]. The genetic diversity of wild plant species is generally unknown, sampling strategies based on predictive models for the capture of alleles across a population with increasing sampling effort, taking into consideration factors such as the population structure and inherent species’ traits (e.g., pollination syndrome) are helping improve previous rules of thumb [58,61]. The needs of the end user (e.g., restoration, plant health research, plant breeding, etc.) must also be considered when developing a sampling strategy, for some uses maternal lines should be banked separately (e.g., UK National Tree Seed Project, [62]). Both seed and living plant collections offer opportunities to add back diversity to wild populations that have had their extant populations reduced [63,64]. 

##### Germination

Germination tests are the most effective method for checking the viability of a seed accession, they also provide a protocol (set of test conditions) for turning the seed back into a plant. At the MSB, all accessions of sufficient size (see below) undergo an initial germination test post banking. This represents a significant task, with over 40,000 species banked from 190 countries each requiring individual germination conditions to be assessed. Re-tests occur every 10 years; however, if longevity is known to be short or seen to be declining, the re-test interval is reduced to 5 years. 

For accessions with an adjusted (see below) seed quantity of >2500 seeds, 50 full seeds are used for the test and up to five initial tests with varying conditions may be performed. This requires an over-sow calculation should the adjusted seed number be lower than the original seed number. For example, if 45 out of 50 seeds were full in the X-ray test (90% potentially viable), a total of 56 seeds (50/0.9 to the nearest whole number) would be required per test to account for potentially empty seeds. The number of seeds per test and number of tests decreases below this accession size, until for accessions with <259 seeds no test is performed. In no instance should more than 10% of an accession be used, this ensures sufficient seeds remain in the collection for monitoring and use over the predicted lifespan of the collection in storage. The MSB’s use of 50 seeds in periodic germination (viability) tests mirrors the FAO recommendation for distribution. It is less than the 200 usually recommended by ISTA, to both avoid excessive depletion of wild species collections, which are generally smaller than those in crop genebanks, and also to make best use of limited staff time. 

##### Anticipating Loss of Viability and Decline of Longevity

The longevity of an accession in storage is dependent on a variety of factors, including species characteristics and genetics, the point of seed development at the time of harvest, post-harvest handling and storage conditions employed. Monitoring of viability in storage is vital, as declines in viability represent a loss of genetic diversity from an accession, and management decisions around recollecting or regeneration will be required. 

The MSB uses analysis of the results of periodic viability testing of accessions to fit survival curves, extrapolation of which can be used to predict when the viability of each accession would reach regeneration level (75% of initial viability, variation from FAO standards, allowing for wild species issues) [14]. Approaching that level would trigger a management decision; and in practice regeneration would be a very rare event, with a request to re-collect in the source country, if possible, being the preferred option. For a significant proportion of the MSB’s seed accessions made since 2000, there are not yet sufficient viability test data points to permit fitting of survival curves, from which to estimate likely regeneration/recollection intervals. The relatively sparse, usable real-time survival data have been supplemented by an on-going programme of comparative accelerated ageing experiments across diverse species (see [65,66]). While the possibility that the causes of death of individual seeds are different under accelerated ageing conditions from those in long-term storage in a seed bank, the data provide a relative ranking of species’ likely storage longevity. These are used to inform decisions on monitoring period (reduced from 10 years to 5 where a species’ seeds are suspected to be relatively short-lived). In addition, accessions from taxa known or predicted to be short-lived under traditional seed bank conditions have a subsample backed-up under cryogenic storage (liquid nitrogen, see below, Figure 1, and [67]), though more research is needed to confirm the expected improved survival at ultra-low temperatures. 

##### Genetic Integrity

The MSB at present does not engage in any direct routine assessment of the genetic integrity of accessions or its decline. With the particular issues attached to regeneration of very diverse species from many countries, we do not engage in regeneration, except in certain circumstances, mostly for UK native species. Instead, so far as is practicable, we rely on the correlation between loss of viability and decrease in genetic diversity; and efforts are focused on making collections in the field of the highest viability, transferring them as quickly as possible to optimum storage conditions, followed by regular monitoring of viability in real time. Recollection from the wild is the preferred method of replacing an accession, from the original population if it still exists, or from an alternative population if that is possible. Analysis of the survival of MSB accessions over periods varying from 10 to >40 years are giving preliminary indications that 80–85% of accessions are not yet showing any detectable loss in viability and thus are assumed to remain at or close to original levels of genetic diversity and representative of the populations from which they were sampled. For accessions that do show a loss in viability, or no initial viability, recollection is recommended, or required. Since 1984, the average number of species recollection requests per annum is 26.

##### Seed Supply

Seeds are available to bona fide individuals representing recognised organisations for non-commercial purposes (e.g., research on seed biology, morphology or germination), as defined by a material transfer agreement through the MSB Seed List [68]. The only collections available for distribution are those with: a verified name; adjusted seed quantity >1050; a germination test within the last 10 years; permission from the donor or project; not covered by CITES; and not held at the MSB under quarantine conditions in compliance with current UK Plant Health Regulations. Up to 60 seeds per accession are supplied, the number depending on the adjusted seed quantity of the accession, following the FAO minimum recommendation (30–50 seeds [20]). However, the standard does not cite specific population genetic research in support of the recommendation.

##### Staffing

Wild species seed accessions at the MSB currently amount to ~97,000, of ~40,000 diverse species; with an addition of around 3000 accessions per annum. Collection curation consists of accessioning/databasing, drying, cleaning, banking, viability testing (initial and periodic), sample distribution, etc.; and needs a team of a Seed Collections Manager plus 16 staff, with a variety of skills and experience: and, unless growth of the collection is to lead to unmanageable backlogs of processing and periodic viability testing, the team needs the addition of a seed collections assistant every 2–3 years.

## 3. Challenges and Prospects

While the importance of living collections and seed banks has been clearly demonstrated, together with the wider work carried out by botanic gardens in this sphere (e.g., outreach and education), there remain challenges to the long-term conservation of plant diversity using these ex situ conservation options. 

### 3.1. Geographic and Taxonomic Biases in Collections

**Figure 3:**
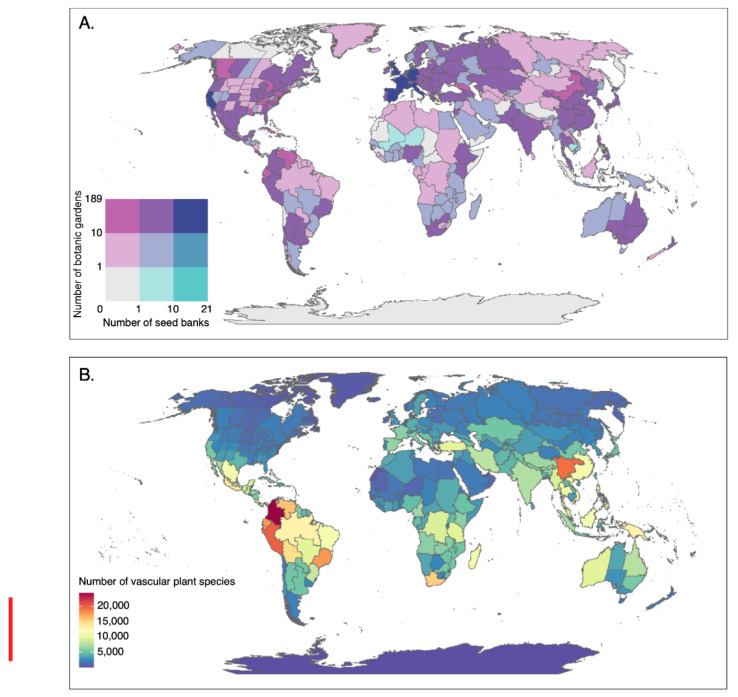
Geographic distribution of (**A**) ex situ conservation capacity and (**B**) centres of plant species richness. Ex situ conservation capacity is represented by the numbers of seed banks and botanic gardens found in each botanical region of the world according to level 3 of the World Geographical Scheme for Recording Plant Distributions (WGSRPD) [69]. Numbers of seed banks per region were extracted from the Millennium Seed Bank Partnership (MSBP) and Botanic Gardens Conservation International (BGCI), whereas numbers of botanic gardens per region were extracted from only the latter. For direct comparison, plant species richness was also extracted for each botanical region (level 3 WGSRPD) from the World Checklist of Vascular Plants [70].

While the number of botanic gardens working in conservation, and the number of seed banks conserving wild species, has greatly increased in recent years, there remains a disparity between the location of biodiverse, threatened habitats and the sites of these conservation centres (Figure 3) [26]. Based on the records in BGCI’s PlantSearch, the distribution of botanic gardens appears disproportionately temperate, with 93% of plant species conserved in the northern hemisphere—mainly in Europe and North America [26]. BGCI’s GardenSearch similarly shows that two thirds of the 551 gardens that provide records for plant conservation programmes are based in high-income economy countries as defined by the World Bank [34]. While this may represent a slightly distorted view, as there are more countries in the northern hemisphere, and gardens located in the northern hemisphere have greatest data sharing capacity, it highlights the need to support and establish botanic gardens in the tropics. This need is emphasised by the finding that an estimated 76% of species not currently held in living collections are tropical in origin [26], and that the majority of the world’s plant conservation collections are located outside the most biodiverse regions, with only a third occurring within the 36 global biodiversity hotspots [34]. 

Seed collecting programmes of the MSB have typically targeted areas of high biodiversity and conservation need, originally in temperate and dry regions and more recently in tropical regions. To date, 71% of MSB partner countries occur within the 36 biodiversity hotspots—but only 27% of countries where the partner is a botanic garden lie within a biodiversity hotspot compared to 78% of those where partners are not botanic gardens, and 76% of countries with multiple partners, both within and outside botanic gardens. However, the disparity between collections-based institutes in the northern and southern hemisphere is still evident within the partnership, with only 18% of MSB partner countries occurring in the southern hemisphere (22% for non-botanic gardens partners, 14% for countries with both botanic garden and other institutes as partners, 0% for countries with only botanic garden partners). When looking at the economy of the MSB partner countries, as defined by the World Bank, a similar pattern emerges, with botanic garden only partner countries occurring in only upper-middle and high income brackets, those with partners within and outside the botanic garden sector also include some lower income countries, and only those countries with partners exclusively outside of botanic gardens also include low income countries. 

In addition to the geographical gaps in collections, botanic gardens and collections-based institutes, there are phylogenetic biases in the species that are conserved in living collections and seed banks. While 30% of plant diversity has been found to be conserved in botanic garden collections, representing 59% of all plant genera, there are certain groups of plants that are less well represented. For vascular plants 93% of families and 50% of genera are conserved, but for non-vascular plants only 5% of genera are held [26]. Furthermore, certain plant families and genera are favoured, not least because of their ability to be readily propagated and grown under prevailing conditions, but also because of their horticultural appeal. Most species cultivated in botanic gardens, particularly larger longer-lived species, are represented by an average of two to three individuals, and plants are often clonally reproduced and shared, meaning that the genetic diversity of the wild species or even population is not represented. In addition, as individuals and not populations are conserved, and the number of individuals that can be housed is limited, genetic bottlenecks can arise [71]. To be effective as conservation collections, 10–100 s of individuals of known wild origin, collected from across the ecological and geographic range of the species, are required [34], highlighting the importance of metacollections.

Furthermore, duplication between institutions is desirable to mitigate for loss due to attrition, pests and disease outbreaks, natural disasters and theft [58].This is one important reason why many botanic gardens have developed conservation seed banks and are linking accessions to form metacollections of a given species or genus. For the latter, it is preferable if the material held represents germplasm from separate collecting efforts of different populations to maximise global coverage and relies on excellent record keeping tracking the wild origins of shared collections.

Of increasing concern are gaps in ex situ collections of plants with known uses, many of which are also threatened. For example, a study of the neglected and useful plants of Mexico found gaps in the conservation of wild edible plants. Although 2598 wild plant species (more than 10% of the Mexican flora) are conserved ex situ as seeds in Mexico, with duplicates stored at the MSB, only 62 seed accessions of 21 species from the most important groups of neglected and underutilized plant species mentioned in the review have been safeguarded in the seed banks [72]. In addition, the lack of coverage of these species in ex situ collections means that the associated research needed for species propagation (e.g., germination requirements, dormancy issues, etc.) at a scale to support agriculture and restoration activities is also missing. 

Although 75% of all embryophyte plant families are recorded as being conserved in botanic gardens [26], it is well known that not all of plant diversity can be stored as living or seed collections (Figure 1, [26,73]). A further issue with seed banks is that the material is in stasis, offering a snapshot of the genetic diversity of a population at the time of collecting. While this is helpful for resurrection studies (e.g., [74]) it means that material is no longer evolving. Living collections have a similar problem; while their plants can adapt and evolve, it is to conditions often outside their native range. 

Small accession size (low seed number) can be an issue for seed banks too when working with very threatened or rare species, which tend to enable only small seed accessions to be made from relatively few individuals. One option to overcome this is to employ multiple-year collecting to increase accession size, or to grow material on from germination tests in order to harvest additional seed (regeneration) or produce tissues (e.g., shoot tips, somatic embryos, etc.) that can be preserved in tissue culture or cryopreserved accessions (Figure 1). Ex situ collections of ‘exceptional species’ often inadequately capture the diversity needed to represent diversity likely to be lost in the wild, even more so that for non-‘exceptional species’. The increasing discriminating power and lowering costs of molecular techniques means that they can be routinely added to conservation workflows to increase the diversity of species within seed and genebanks, and for end use in translocation (movement of plants between different sites) and restoration [75]. This is particularly helpful in situations where germplasm is held in living collections, where each plant held represents significant cost to the managing organisation, minimising duplication, while holding high diversity. For example, at the Australian Plantbank, *Rhodomyrtus psidioides* and other critically threatened rainforest species, are being held as living collections, in nursery-held pots, in gardens, field genebanks and in tissue culture [76]. These can be seen as intermediate steps to the lower-cost long-term ex situ conservation goals of securing appropriately diverse germplasm of these species in seed banks, cryostorage and as important elements of metacollections [22,23].

### 3.2. Cryopreserved Plant Collections

For conventional seed banking, an increasing challenge is the inability to store material from ‘exceptional species’ for the long-term [11,12]. While living collections can overcome this difficulty (Figure 1), issues around the amount of genetic diversity conserved and the possibility of genetic erosion, hybridisation or problems associated with pathogens and pests remain [77,78,79]. Cryopreservation is increasingly recommended as a solution (Figure 1), enabling the long-term preservation of a diversity of plant materials (e.g., cells, spores, pollen, shoot tips, seed embryos, whole seeds) and taxa (from algae to bryophytes, ferns, cycads and orchids) and the storage of relatively comprehensive genetic diversity of the population sampled on a relatively small space [12]. Plant cryopreservation is based in the use of ultra-low temperatures (typically those provided by liquid nitrogen, <−130 °C) and often chemical protectants to preserve cells and tissues without the formation of lethal intracellular ice. There are different approaches that can be used which are mainly based on the vitrification (i.e., ’ice-free‘ solidification) of the cell cytoplasm while protecting its physicochemical properties and the structural integrity of tissues [12,77]. 

However, unlike conventional seed banking, plant cryopreservation does not have a universal formula that can be used to preserve a wide range of plant taxa and tissues, and cryopreservation protocols must often be developed and adapted at the species or variety level [20] (Figure 1). In addition, the level of success of many protocols is lower than that applied to germination standards set for conventional seed bank collections. For example, a cryopreservation protocol is considered successful when regeneration is accomplished in at least 20–40% of the preserved samples [80,81], for long-term conservation seed banking storage is considered successful if levels of germination are above 75% initially and do not drop below 85% of initial test levels on subsequent testing [20]. These discrepancies, in both the lack of a universal method for plant cryopreservation and in the levels of initial percentage of plant regeneration between cryopreserved plant collections and conventional seed banking, have often been a barrier for the establishment of cryopreserved collections of wild species within seed banks. However, this has not been the case for crop plants [12,82] and we think similar standards of success should be applied to wild and threatened ‘exceptional species’ [83]. In this regard, cryopreserved plant accessions of wild species can be viewed like the elements of metacollections described in a previous section of this paper. For example, in the case of the rare Sinkhole Cycad, *Zamia decumbens*, indicated above, over 60% of known genetic diversity for this cycad could likely be preserved by combining the curation, in vitro culture and/or cryopreservation of 30 maternal lines (35% of known genetic diversity for this cycad) and the preservation of pollen from 100–200 individuals. This metacollection of cryopreserved, in vitro cultured and whole plant germplasm would conserve a high genetic diversity of this rare cycad in a way that, if seed banking is not easy, living metacollections alone would find challenging.

Cryopreserved plant accessions are relatively common for the long-term conservation of certain crop species that are propagated vegetatively or have desiccation sensitive seeds [77]. However, cryopreserved collections of wild plant species are not common but are increasingly being considered and created within conventional seed banks and botanic gardens. Examples include the CryoBiobank of the Cincinnati Zoo and Botanical Garden, created in the late 1980s and holding the oldest, largest and most diverse collection of cryopreserved plant cells and tissues [84], the MSB, which cryopreserves short-lived seeds and spores, the USDA/ARS National Laboratory for Genetic Resources Preservation that hold seeds and spores of the CPC network, and diverse Australian genebanks [85] (see Box 1). However, we need to expand the number and scale of these types of collections, particularly in tropical areas, where the proportion of species that cannot be banked using traditional seed banking is larger [12,84,86]. Cryopreserved crop collections offer a great source of knowledge not only in the techniques that can be applied but also in solutions for challenges that arise from the management of globally cryopreserved collections [87]. Some historical cryopreserved wild plant collections have provided data to evaluate not only the costs and challenges of preserving wild plant species in vitro and stored in liquid nitrogen, but also on the stability and longevity of the preserved samples [12,84,88]. 

Nevertheless, there are some aspects to consider if we want to increase the number and scale of cryopreserved plant collections of wild species. Firstly, the ’fear‘ of using liquid nitrogen technologies needs to be reduced, as this is often the first barrier to the development of basic cryopreserved plant collections across conservation institutions. For example, cryopreserved collections for short-lived desiccation tolerant seeds, fern spores, and desiccation tolerant pollen can be created with minimal investment and training, as dry seed, spore and/or pollen collections can be stored in liquid nitrogen with relatively low technical requirements [67]. Secondly, conventional seed banks and botanic gardens need to invest in infrastructure and specialised training to increase the taxonomic and geographic variation of cryopreserved collections of wild plant species (the scale of the investment will depend on the scale of the cryobank desired). Thirdly, cryobiotechnological research needs to be increased if more species and tissues are to be successfully cryopreserved [12,83,84,85,89,90,91]. Fourthly, we must strengthen networking between wild species genebanks and crop genebanks to facilitate the preservation of wild species collections at the regional level in their crop genebank cryobank facilities, particularly in tropical areas where funding, a stable supply of liquid nitrogen and training for the development of wild species cryopreserved collections may be challenging [84].

Box 1 **Meeting the challenges of seed banking and conservation of ‘exceptional species’ in Australia.**

Australia has a large, diverse flora and many endemic species. The negative effects of climate change—such as decades of frequent drought and bushfires—coupled with habitat loss, rapid spread of invasive weeds and disease, have resulted in an increased level of threat to native flora [92]. The unprecedented scale and intensity of the ‘Black Summer’ fires of 2019/20, burning more than 10 M ha of land in south-eastern Australia across 11 Australian bioregions and 17 major native vegetation groups [93], has undoubtedly brought many species closer to extinction. Botanic gardens are being activated to help monitor post-fire recovery and to collect germplasm for ex situ conservation.

There are presently ten conservation seed banks in Australia, mainly in botanic gardens, that hold 68% of threatened flora represented by at least one accession [92]. Efforts have largely focused on dryland species, due to the expectation of desiccation sensitivity of seeds of rainforest. Several studies have explored the seed storage potential of Australian rainforest flora [49,94] and these results were combined with other data sets to develop a key for determining the seed storage potential of untested rainforest species [49]. This key will help us to understand the complex biology of ‘exceptional species’ and recognise species that need conservation efforts beyond the traditional seed bank.

A number of Australian crop wild relatives fall into the exceptional category, including *Citrus*, *Syzygium* [95] and *Macadamia* species. *Macadamia* is Australia’s only indigenous crop grown at large scale, and current efforts are focusing on the twin challenges of securing the remaining germplasm of the four *Macadamia* species in the wild (which are all threatened), while ensuring the availability of material for inclusion in breeding programmes. Alternative conservation methods such as tissue culture and field genebanking are available for such species [96]. The further development of cryostorage techniques for ‘exceptional species’ conservation is an increasing focus of collaborative research due to the great potential of this technique as a long-term conservation option [85,97,98].

The recent environmental disasters in Australia have greatly increased the imperative for ex situ conservation of all species, but particularly for those of fire-impacted east coast rainforests, including the relictual Gondwanan rainforests. These forests rarely burn, and the species are often poorly adapted to fire. The fires may have left these forests ‘susceptible to regeneration failure and landscape-scale decline’ [93]. Many species are already under pressure from habitat loss due to clearing, the effects of invasive weeds and diseases. An added, looming existential threat to rainforest Myrtaceae species, is the recent incursion of Myrtle Rust fungus (*Austropuccinia psidii*). This disease has spread rapidly to more than 358 species in Australia since its unfortunate introduction in 2010 and has been found more recently in New Zealand. Myrtle Rust is decimating a number of once common rainforest species, such as the Scrub Turpentine (*Rhodamnia rubescens*), and Native Guava (*Rhodomyrtus psidioides*), with imminent annihilation expected for a number of species [99]. If the plants are not killed outright, the disease often affects the flowers and fruit and therefore collecting of seeds from the wild is usually not an option. The disease can be controlled in cultivation; however, progress of the disease may outpace germplasm capture for ex situ conservation such is the scale of the problem [76,100].

### 3.3. The Importance of Networks

Developing and maintaining active networks of institutions focused on a shared goal can be a significant tool for nations to meet their commitments to international plant conservation and restoration targets [101]. Well established networks aimed at tackling plant conservation issues exist at different levels: locally, nationally, regionally, and globally. Networks operating at the national level often have a highly targeted approach to plant conservation. An example is the Mexican native species Biodiversity Nurseries Network (REVIVE), implemented jointly with the Seed Reserve (RESEM) in the Veracruz State. Their mission is to increase the diversity of native species growing in nurseries for restoration purposes. Initially most nurseries only worked with native *Pinus* species, but REVIVE now have more than 200 native species from different Mexican ecosystems available as seedlings or seeds for distribution [102]. National networks can also feed into regional ones. ENSCONET is a regional network of institutes with an interest in native species conservation through seed banking that includes the Italian seed banking network (RIBES) [103] and part of the Spanish seed bank network (Red Española de Bancos de Germoplasma de Plantas Silvestres) [47] and Mediterranean network (GENMEDA) [104] as part of their current membership. Collectively, the membership has contributed to ensuring 62.7% of European threatened species are in long-term conservation [105]. 

Global networks have the greatest diversity of associated organisations and have the potential of having the greatest global impact. Under the umbrella of the MSBP, botanic garden seed banks, agricultural genebanks and forestry seed centres are brought together with a shared purpose, enabling the development of more collaborative and complementary conservation programmes. Similarly, BGCI’s Global Seed Conservation Challenge (GSCC), a network of over 200 botanic gardens involved in seed banking, supports seed banking through provision of training, resources and funding while challenging botanic gardens to conserve more threatened species in seed banks. Since 2015 the number of taxa conserved as seed added to PlantSearch has doubled. Through the GSCC fieldwork fund, 120 species have been collected, including 45 threatened with extinction. These global networks and consortia can make a significant contribution towards global conservation and restoration targets but do require significant levels of resourcing to be effective at a global scale. 

The existence of a maintained network can increase reactivity and dynamism amidst political and environmental instability. Climate change is leading to an increase in extreme weather events, including drought and related wildfires, hurricanes, and flooding. In 2012, the US endured several environmental disasters including the burning of two million acres of sagebrush in four western States, and widespread damage to native plant communities responsible for stabilizing soils and filtering water on the East Coast by Hurricane Sandy. These events led to the creation of the National Seed Strategy for Rehabilitation and Restoration to provide a more coordinated approach and response to these large-scale events [106]. Ecological restoration is often constrained by a lack of the large quantities of seed required. The Strategy focuses on the establishment of a nationwide network of native seed collectors, farmers and growers, nurseries and seed storage facilities to supply adequate quantities of appropriate seed, together with a network of restoration ecologists. The vision of the strategy is ‘The right seed in the right place at the right time’. A progress report in 2021 showed 380 partners are involved in the resulting network and have together invested $167 M in the programme [106]. 

More recently, the 2019 ‘Black Summer’ bushfire season impacted 67–83% of globally significant forests and woodlands of Australia, and decimated >50% of known populations or ranges for over 800 vascular plant species native to Australia (see also Box 1) [93]. Australian botanic gardens have long had a strong focus on the conservation of native and, particularly, threatened species. This was fostered by the formation of the Australian Network for Plant Conservation in the early 1990s, followed by various partnerships with the MSB from the late 1990s, which in turn enabled the establishment of the Australian Seed Bank Partnership (ASBP) [107]. These networks and partnerships supported the development of seed banking capacity in each State and Territory, and firmly placed botanic gardens as providers of plants and services for conservation of Australian native species. The ASBP enabled a rapid response post-fire in the form of habitat assessments and collecting seeds from remaining individuals found in refugia, as well as long-term monitoring of habitats of affected species over the coming years. ASBP is working not only with plant conservation consortia across Australia but remains part of the global network of the MSBP. This continued partnership provides further security for the Australian seed collections, with over 9000 Australian species duplicated to the MSB over the past 20 years and forming an integral part of their collections, with the potential for repatriation when required. This ability of networks to enable reactivity and dynamism in an ever-changing world will be increasingly important for plant conservation and responses to the biodiversity crises in the coming decades.

In relation to cryopreservation of ‘exceptional species’ plant cryobanks are well established in many national and international crop centres, such as Bioversity International, the International Center for Tropical Agriculture (CIAT), the International Potato Center (CIP), the International Institute of Tropical Agriculture (IITA), the World Agroforestry Centre (ICRAF), the Global Network on Cacao Genetic Resources Conservation and Use (CacaoNet), the USDA/ARS Agricultural Genetic Resources Preservation Research, the Leibniz Institute of Plant Genetics and Crop Plant Research (IPK), where vegetatively propagated or desiccation sensitive seed crops are preserved in liquid nitrogen [77,87]. Some of these cryobanks are well interconnected through global networks and initiatives such as the CGIAR [77,87]. Similarly, for the forestry sector, nursery stands of economically important tree species are general practice across many agroforestry centres, allowing for the use, conservation, improvement, and distribution of germplasm [108]. Coordinated networks for the conservation of ‘exceptional’ wild species are less prominent; however, there are notable examples. At a global scale, BGCI in collaboration with Valerie Pence at the Cincinnati Zoo and Botanical Gardens is working to link knowledge, resources and projects globally to conserve threatened ‘exceptional species’ in a systematic way [109]. Within this collaboration they are promoting the creation of an Exceptional Plant Conservation Network (EPCN), that aims to share resources, including a list of ‘exceptional species’, information and links to information on the species and alternative conservation technologies, ways to link to other researchers working with ‘exceptional species’, as well as other supplemental information [110]. The EPCN is planned as an open resource that will benefit from the input of all researchers who are working on the conservation of threatened ‘exceptional’ plants. Moreover, BGCI’s Global Conservation Consortia coordinates networks of institutions and experts towards the conservation of priority threatened plant groups, such as maples, oaks, magnolias, and dipterocarps. These networks help to develop and implement comprehensive strategies for in situ and ex situ conservation efforts (including cryopreservation) and dissemination of species recovery knowledge. At the national scale, networks such as the CPC in the USA, promote research and the use of tissue culture and cryopreservation techniques as alternative storage methods to conventional seed banking for the conservation of threatened ‘exceptional species’ [57].

### 3.4. Data Management and Access

Data sharing is key for enhancing global plant conservation collaborations, enabling exchange of knowledge across networks and the establishment of metacollections. For both living collections and seed bank accessions, the associated data, gathered through the collecting, processing and/or growing activities, represents a wealth of potential for collection management, research, restoration and conservation action [111,112]. Typically, data shared by botanic gardens is related to taxonomy, distribution, conservation status, plant availability in gardens, uses and a brief description of the plant. Data captured by seed banks is generally focused on seed traits such as seed storability, viability and/or germination, morphology, collection location, and seed availability. 

A challenge for data sharing, particularly for wild species seed banks, is the incompatibility of different data management systems across botanic gardens and seed banks. Data sharing for crop species is more advanced in relation to unifying accession and trait data under an open access system (e.g., Genesys [113]). The MSBP network goes some way to tackling this issue through the development of the MSBP Data Warehouse [114]. Developed in 2015 as an online resource for partners, this platform aggregates data from partner’s in-country collections with the duplicates held at the MSB. Currently, the MSBP Data Warehouse holds data of 230,166 seed accessions, 2074 X-ray images and 220,295 germination data, and partners can access the majority of this seed related data from across the global network. It also provides links to other RBG Kew online resources such as Plants of the World Online (POWO) and the Seed Information Database (SID). Processing data for duplicate accessions is repatriated through the system, highlighting accession quality and enabling reflection on existing processes to aid future collecting. 

Alongside the use of networks as a hub for data and knowledge exchange, the development of shared network systems can be a way of accelerating institutional data onto globally accessible platforms. In 2005 ENSCONET developed ENSCOBASE [115], an open access database where members of the network can upload data on native European wild species within their seed bank. Publicly accessible platforms such as ENSCOBASE and PlantSearch can be used to measure progress towards international targets such as the GSPC by tracking collections of threatened species [105]. They also connect accessions directly to conservationists, educators, horticulturalists, researchers, policy makers and many others who are working to conserve and understand plant diversity, and data can be used to prioritise conservation of threatened species not held in ex situ collections. This prioritisation can be implemented by individuals, organisations or networks at the local, national, regional or global level depending on the species’ distribution. The number of seed banks uploading data to PlantSearch has doubled in the last five years; however, only around 80 of the 355 botanic gardens with seed banks upload their seed accession data to PlantSearch in addition to information on their living collections. Institution level resourcing to facilitate upload of data is an issue common to many conservation-oriented data sharing platforms—however, the more data these platforms contain, the better-informed conservation actions will be. 

The MSB Data Warehouse, ENSCOBASE and PlantSearch are among the very few examples of initiatives to share data across and beyond the botanic garden network. A wide range of different systems are used by botanic gardens to maintain accession-level data on the plants in their collections, but unlike the plant genetic resource sector, data sharing and the adoption of common data standards has not yet been a priority. While some botanic gardens (e.g., RBG Edinburgh) provide on-line access to collection catalogues, this tends to be the exception rather than the rule. Legitimate concerns about potential theft keep curators of living collections from sharing their full catalogues. However, an initiative from the German Botanic Garden Network (gardens4science) aims to develop a data portal giving accesses to the local databases of the living collections of more than 10 gardens, starting with bromeliads and cacti [116].

Sharing data relating to species that are highly threatened or have the potential for exploitation also presents a challenge for seed banks. Accession data typically have locality and use documented within databases, and whilst this provides opportunity for use in research and conservation, it can lead to misuse and may exacerbate illegal trafficking of material for commercial gains [117]. The MSBP Data Warehouse ensures compliance with agreed use of associated data from donating institutions in two ways. The first is by sharing an online view containing ~85% of the offline database, with any requests for data sensitivity accounted for. The second is the ability to restrict locality information through ’fuzzy mapping‘, where coordinate data can be restricted, or resolution decreased on the interactive map. ENSCOBASE uses an alternative solution, and only provides location data at the country and biogeographic region level. 

Language can be a limiting factor in the sharing of information and knowledge. Although English is generally accepted as the language of science, the greatest plant diversity lies in regions where English is not the native language, and these are also the areas experiencing the greatest threats to plant species survival [118,119]. The use of multi-language tools and ensuring that information generated for a specific location’s flora is available in the local language will help relieve this issue. There are good examples of developing such material: the ENSCONET seed collecting manual is available in nine European languages; and the ColPlanta website [120] for Columbian plant and fungi information is accessible in English and Spanish [121]. Similarly, global aggregators such as the Global Biodiversity Information Facility (GBIF) [122] have the capacity to switch to a variety of commonly used languages. 

Finally, we must also acknowledge the global disparity to internet access and the impact that this has on successful data exchange, particularly for those within global networks. Areas where there are high biodiversity and associated threats to plants, such as much of Africa and Papua New Guinea sit relatively low in the rankings of internet users per population size [123]. For these key areas, the development and maintenance of national and/or regional networks remain important, as they serve as a way of ensuring continued support and knowledge exchange across multiple players in plant conservation within high biodiverse ecoregions. The inclusion of expansive knowledge and technology transfer programmes that enhances capacity of local collections alongside global repositories can also mitigate the issue of access, for example, the various standard and bespoke training programmes run by BGCI and MSB. 

### 3.5. The Importance and Challenges of Material Sharing

We have already articulated the importance of duplicating seed collections at two geographically distinct seed banks and joining living collections in metacollection strategies. This insurance policy is increasingly important as environmental change becomes less predictable and impacts greater areas. For many critical plant species that are in living collections, a singular locality can dramatically increase the risk of a collection being compromised either through total loss or gradually, through genetic erosion. Furthermore, use of seed material for research can also contribute towards species conservation [112] and to finding solutions to global challenges, such as food security [124]. 

There are various challenges in relation to sharing of material globally. One relates to colonial histories and biopiracy, where historical imbalances have led to a lack of trust in material sharing, particularly at the international scale, potentially to the detriment of global plant conservation. Limiting access to physical material and associated data can hamper research progress, potentially impacting the long-term conservation of endemic floras. As plant species’ distributions straddle national boundaries, these limitations can also impact the conservation of species with regional or global distributions, making a comprehensive assessment of their risk of extinction and overall management difficult. 

In 1993 we witnessed the first step change in recording consent through the ratification of the Convention on Biological Diversity (CBD) [125]. The ratified members of the CBD recognise the sovereign right of countries over their genetic material and any sharing of materials across national borders must take place in the context of PIC and under an agreement on the terms of transfer of the material, including any subsequent access and benefit arising from its use. The Nagoya Protocol provides a legal framework for the access and benefit sharing of biological diversity [27]. Benefit sharing negotiated between parties can include, but is not limited to, access to accessions and associated data, augmentation of national collections, transfer of technology, training, joint research activities and in the case of commercialisation, monetary exchange. 

The implementation of the Nagoya Protocol (currently ratified by 132 countries) requires botanic gardens in both provider and user countries to understand the modalities involved in collecting and storing plants and seeds outside their national boundaries. In Europe and beyond, many gardens have joined the International Plant Exchange Network (IPEN) and/or endorsed the Kew Principles on Access and Benefit Sharing. These initiatives include Codes of Conduct which guide how material in collections can be used and shared in line with the Nagoya Protocol. The principles of Access and Benefit Sharing also apply within countries and have shone a light on ‘ownership’ of natural resources, especially those which occur on land owned or managed by indigenous communities. In Mexico for example, the national botanic garden network has developed a Code of Conduct and best practices for collecting seeds with indigenous communities, and a similar code is under development in Australia [126]. 

The establishment of a policy group at RBG Kew with legal expertise to develop, maintain and record agreements has ensured its compliance with the CBD and CITES. The majority of MSB partnerships are developed through bilateral agreements, where the terms of material use (i.e., Material Transfer Agreement) are clearly outlined. Continued communication between the two acting parties ensures benefits are transferred (e.g., germination protocols) and PIC is sought for third party use. Exceptions exist, for example, the Adapting Agriculture to Climate Change project [124] was governed through the Multilateral System (MLS) of access and benefit-sharing under the International Treaty on Plant Genetic Resources for Food and Agriculture (ITPGRFA). Negotiations for this type of relationship require further trust-building and ought to incorporate the use of the ABS clearing house as part of the project to ensure compliance. 

Bilateral (or multilateral) agreements are typically made at the national level, and so tend not to capture the diversity of stakeholders required to deliver highly impactful conservation outputs. The inclusion of indigenous and/or local communities in relation to access (i.e., of land, knowledge and material) and benefit sharing (e.g., monetary/non-monetary and national/international) is an increasingly important aspect to consider [127], particularly in countries where well-established networks exist. Examples include Australia, New Zealand, Canada, and the United States. An added challenge is the practical management of data relating to indigenous knowledge, a subject currently actively debated within the museums and humanities sectors [128]. 

Living material (e.g., live plants and seeds), have the potential to carry a variety of bacteria, viruses and fungi as part of their microbiome [129,130]. The relationship can be beneficial (i.e., required for germination or growth), commensal or pathogenic. Therefore, sharing of material will inevitably carry some level of risk, notably with regards to the introduction and spread of novel pathogens. Since its emergence from commercial nurseries and plantations in South and Central America, the pathogen responsible for Myrtle rust (*Austropuccinia psidii)* has expanded its international range rapidly, spreading into the Australasian continent within the past decade and affecting native populations of Myrtaceae [131,132,133]. The World Trade Organisation Agreement on the Application of Sanitary and Phytosanitary Measures [134] provides the international regulatory system for plant health and aims to prevent the introduction and spread of harmful diseases or pests. Within this agreement are a set of reference standards on which nations can build a legal framework that relates to plant health and trade. For example, the EU Plant Health Regime provides a framework governing the movement of plants from non-EU countries. Import of some species that are deemed high-risk are completely banned, whilst others require accompanying phytosanitary certificates [135]. Aside from selected high-risk material (e.g., *Malus*, *Aegilops*, etc.) requiring ‘plant passports’, plants or plant products can move freely within EU member states. Therefore, any changes within the political landscape, for example, the UK’s exit from the EU, will inevitably have an impact on the ease of material access and sharing. Additionally, processes of disease and pest screening prior to issuing phytosanitary certificates can be flawed or limited by the capacity and effectiveness of a country’s screening measures [136]. The type of material being shared will also need to be considered as the level of regulation can vary greatly (e.g., pollen versus seeds). 

While seed is generally considered to be relatively ‘clean’ with respect to pests and diseases, and its movement is generally less restricted by phytosanitary restrictions than other types of plant material, seed collected from wild populations may present a ’trojan horse‘ for transmission of pathogens. Recent research on seeds of African eggplant wild relatives found the seed contained potato spindle tuber viroid, both a first finding for this host and a first record in several countries. Like seeds, pollen international exchange is considered safe, as harmful pests and diseases are rarely transferred through pollen [77]. However, pollen may be a vector for viruses and other pathogens [137] and specific phytosanitary restrictions have been placed in some plant groups and countries (e.g., *Citrus* in the USA [138]). That germplasm collections may be storing diseased material poses a downstream phytosanitary risk to the collections, species conservation, or future breeding research. 

Considering the challenges of sharing material globally, including constrains imposed by the policy environment, plant conservation efforts must include building capacity in the country of origin, notably in biodiverse regions. One example is provided by the Meise Botanic Garden in Belgium, whose scientists have been studying the wild diversity of *Coffea* in Central and West Africa for almost 25 years. In order to ensure the conservation of important diversity in the Democratic Republic of Congo, Meise Botanic Garden has trained a network of local botanists, ensured the rehabilitation of historic field and herbarium collections and supported the collection, conservation and evaluation of newly described plant species in ex situ collections [139]. The biological constraints of the species and/or seed itself can mean that transporting germplasm may be less favourable than storing it locally. Maintaining or developing networks within countries, including with the agricultural sector that often has cryostorage facilities, will enhance the conservation potential for seeds that are short-lived and/or desiccation sensitive. 

## 4. Plant Conservation in the Wider Context

### 4.1. Integrated Conservation

It has been said that there is no technological reason that any plant need go extinct [26]; however, it is also recognised that seed accessions have a limited shelf life, and that the size and usefulness of accessions will decline over time as they are used for activities such as research, reintroductions, or periodic viability monitoring. Neither seed banks nor botanic gardens, even with support from cryopreservation, are a surrogate for functioning ecosystems, and there will always be a need to preserve plant diversity in situ. Integrated conservation, where botanic gardens and seed banks help ensure plant diversity conservation through outreach and engaging with in situ work, is vital. The following case studies highlight how the knowledge, skills and collections in botanic gardens and seed banks are helping address some of the global challenges facing humanity by halting biodiversity loss, increasing food security and supporting livelihoods. 

### 4.2. Agrobiodiversity—Contribution to Global Food Security

Concern about the impact of climate change on global food security and biodiversity, coupled with continued population growth, has driven initiatives to diversify our food sources and build crop resilience [140]. 

The Adapting Agriculture to Climate Change, or Crop Wild Relatives, Project (2011–2021), managed by the Global Crop Diversity Trust and RBG Kew, and funded by the Government of Norway is such a project. Crop wild relatives (CWR), the distant wild ‘cousins’ of domesticated crops, contain genetic traits that can potentially be harnessed through crop breeding techniques to create climate and pest-resilient crop varieties. The unavailability of these vital CWR plant genetic resources for food and agriculture (PGRFA) to crop breeders due to their insufficient conservation in ex situ collections [141], resulted in this concerted international effort to collect the wild relatives of 29 of the most important food crops from across the globe. Occupying a unique position of experience in the ex situ conservation of wild species seeds, the MSB played a pivotal role as the global duplicate repository, via the use of Standard Material Transfer Agreements (SMTA), for almost 4000 seed accessions of over 240 taxa collected by institutional partners in 21 countries. In addition, by making use of Kew’s vast herbarium, seed collecting guides for each CWR project country were produced. Training in seed collecting and conservation was also provided at Kew and in-country to 174 individuals associated with the project.

In addition to safeguarding CWR seed collections in the countries of origin and at the MSB, the final component of the project was to distribute small samples (typically 100 seeds) of each accession to specialist CGIAR-affiliated genebanks around the world for incorporation into research programmes, investigating traits within CWRs that confer resilience to abiotic and biotic stresses. As such, the CWR project has by necessity brought the MSB’s expertise of wild species seed conservation and the CGIAR genebanks’ focus on improved crop varieties together with the common goals of improving global food security through the sustainable use of plant genetic resources in the face of climate change, improving human nutrition and health and reducing poverty.

Many important crop species cannot, however, be stored in traditional seed banks. The National Tropical Botanical Garden in Hawaii is host to the Breadfruit Institute which includes the largest assemblage of breadfruit cultivars in existence. The Institute is using the knowledge acquired by more than 30 years of conserving and studying breadfruit to plant trees in tropical countries for food and reforestation, provide economic opportunity, and to educate the public about the benefits of growing—and eating—this underutilised crop. More than 300 breadfruit trees are conserved in the living collection and, working with its partners, the Institute is sending micropropagated breadfruit trees to tropical countries worldwide. Since the launch of the initiative in 2009, more than 100,000 breadfruit trees have been sent to 44 countries. The breadfruit collection includes accessions from 34 islands across the Pacific including some cultivars that are now rare or vanishing in their homelands. The geographical scope of the collection also includes accessions from Indonesia, the Philippines, Seychelles and Honduras.

### 4.3. Supporting Food Security and Livelihoods

Throughout human history, wild edible plants (WEP) have been important to rural communities [142,143,144,145], but wild populations of WEP are under threat globally [146]. In the South Caucasus, Kew collaborated with long-standing partners of the MSBP, the National Botanical Garden of Georgia, the Institute of Botany Ilia State University of Georgia, and Nature Heritage NGO of the Republic of Armenia to deliver a 3-year Enhancing Rural Caucasian Livelihoods through Fruit and Nut Conservation project funded by the Darwin Initiative. Due to the strong intrinsic link between plants and people in the Caucasus, this project ensured the conservation of wild species without jeopardising the livelihoods and food culture of the target and surrounding communities. 

Through a multidisciplinary project team, consisting of botanic gardens (and their associated seed banks), local NGOs, social scientists, research universities and community leaders, the project covered five main themes: engagement; in situ and ex situ conservation; research; and training. 

Two communities were engaged with the project, one in the south of Armenia and one in the north of Georgia. Community-led steering groups oversaw various project activities, maintained a participatory approach throughout the project’s lifetime and provided a legacy after the project end. An awareness campaign on the importance of plant conservation and sustainable harvesting led by in-country project members supported by a steering group reached 60% of communities that utilise the harvesting landscape. Through community member project interviews the key WEP harvested were identified together with their use and the level of perceived importance of these products. Thirty native fruit and nut plants were brought into cultivation in three community-run orchards, alleviating overharvesting of wild populations whilst simultaneously providing income for local families. Training on how to propagate and care for the plants was led by local horticulturalists based at the relevant botanic gardens. 

Georgian and Armenian conservationists received IUCN Global Red List assessment training, enhancing the capacity for in situ conservation activities in-country. The training and close collaboration with national herbaria enabled the assessment of 20 fruit and nut species that are endemic to the region. A mixture of species used by local communities and their threatened wild relatives were targeted for seed collection and conservation: 193 seed accessions from 119 different fruit and nut species were conserved and duplicated to the MSB. 

The project aimed to narrow the knowledge gap of WEP and build research capacity within the target countries through engaging two local MSc students. Collaboratively developed research topics revolved around key edible species that are frequently used locally, but rarely investigated (*Rosa* in Armenia and *Prunus* in Georgia). By utilising the expertise both in-country and at RBG Kew, the students were trained in various aspects of plant science, from seed conservation to genomics.

Through the project, the team began to have a better understanding of the way the local landscape is used by local communities, and subsequently the needs for its continued conservation. An important aspect that was outside the scope of the project was studying the impact of commercially driven collectors originating from outside the local area, who can have a significant impact on overall biodiversity at the landscape level if their activities are not conducted sustainably [147]. To explore this further, the partnership will need to develop sustainability training at a commercial scale (e.g., the FairWild model) and expertise in supply chain valuation. Building cryopreservation capacity, both in-country and at the duplication site, for wild edible species that have seeds that are short-lived (e.g., *Corylus* spp.), intermediate (e.g., *Fagus* spp.) and/or recalcitrant (e.g., *Quercus* spp.) would also greatly benefit long-term conservation of these species [67]. 

The Kew-led Useful Plants Project was implemented on the ground over two phases (2007–2010; 2011–2015) under the MSBP with the aim to enhance the capacity of local communities to successfully conserve and sustainably use important indigenous plants in Botswana, Kenya, Mali, South Africa, and Mexico [148]. The project brought together Kew scientists and a wide range of collaborators from different disciplines, involving botanists, horticulturalists, agronomists, and foresters, who worked closely with rural communities, local authorities, and schools utilising participatory techniques. A scientific approach was applied throughout the main components of the project: selecting useful plant species; ex situ conservation; propagation and planting; and supporting people’s livelihoods. Most of the species were reported to have a medicinal use (878) or to be used as food for humans (615) and materials (427). In Africa, prioritised useful plants included: the iconic multipurpose *Adansonia digitata* (Baobab) and the highly valued *Senegalia senegal* (Gum arabica); food plants *Schinziophyton rautanenii* (Mongongo tree) and *Tylosema esculentum* (Morama bean) used in Botswana; the multipurpose timber tree species *Melia volkensii* in Kenya; and several species of columnar cacti and agaves in Mexico used for their edible flowers and fruits [149]. This project has achieved an impact on plant diversity conservation through seed banking of priority useful plants, with 1271 seed lots banked in-country and 952 duplicated in Kew’s MSB. Research on seed germination helped support plant propagation activities, and facilities were set up or improved at the local level for the conservation and propagation of the prioritised species. Training and knowledge in seed conservation and plant propagation were also provided to rural communities while facilities were set up or improved locally. Two hundred and sixty-seven species (76,389 seedlings) were planted in community gardens for direct use, 59 of which (seeds/seedlings/part of plants or their plant products) were promoted for income generation in rural communities through workshops and marketing events. Finally, the project supported local education through the establishment of school gardens and increased knowledge on the conservation and sustainable use of native species and by tutoring undergraduate and postgraduate students. This project highlighted the importance of applying an ‘holistic approach’ to address the dual objective of biodiversity conservation and contribution to improved livelihoods in the local communities [148].

### 4.4. Restoration and Reforestation

The important role that botanic gardens can play in supporting ecological restoration through the provision of scientific knowledge and plant material has been well documented [150]. In GardenSearch, 329 institutions are listed as having a plant reintroduction programme, and 226 are involved in restoration ecology research. The Ecological Restoration Alliance of Botanic Gardens includes 49 members. Here we consider five examples which show how the integrated plant science and conservation expertise of botanic gardens and associated seed banks are helping deliver practical, on-the-ground solutions to plant and habitat loss. 

The **UK Native Seed Hub** (UKNSH), established in 2011, was conceived as part of RBG Kew’s response to an independent review commissioned by the UK Government, of England’s wildlife sites and the connections between them, entitled ‘Making Space for Nature’ [151]. The report proposed a long-term strategy, to 2050 and beyond, for conservation in England based on rebuilding nature, fragmented and degraded due to human activities, at a landscape scale, by creating coherent and resilient ecological networks that would link and expand existing habitat patches with buffer zones, wildlife corridors and areas of active restoration and habitat creation. 

A significant constraint to effective conservation and habitat restoration is the limited availability of known-origin, high-quality, genetically diverse seeds and plants [112]. The MSB, through its comprehensive UK seed collections coupled with its scientific and technical expertise, was well placed to address this shortfall. Since 2011, the UKNSH has embraced this role by providing plant materials (seeds and plug plants), applied research and technical assistance to over 63 conservation projects, and has partnered with more than 41 organisations in the UK from both the public and the private sector.

One of the chief aims of the UKNSH from the outset was to support the goals outlined in the Lawton Report (2010) to rebuild nature on a landscape scale, enhancing and strengthening ecological networks [151]. Eight years on from the publication of that report, while evidence of success in terms of landscape scale conservation nationally is unclear and at best mixed [152,153], the UK Government published a broader 25 Year Environment Plan [154], which included a commitment to develop a Nature Recovery Network, launched in 2020, to expand, improve and connect a national network of wildlife-rich places across towns, cities and the countryside. Going forwards the UKNSH, in line with Kew’s Manifesto for Change 2021–2030 [155], will continue to support these aims and seek further opportunities to work with the government, landowners and managers, businesses, local communities and conservation organisations.

**China** is home to 10 percent of the world’s total plant diversity, some 30,000 higher plant species [156]. However, there are many threats to China’s native flora including rapid socio-economic development, climate change, habitat conversion and unsustainable use of native species. The country is a key region for BGCI’s mission to mobilise botanic gardens and engage partners in securing plant diversity for the well-being of people and the planet. BGCI launched its China Programme Office (hosted by the South China Botanical Garden Chinese Academy of Sciences, Guangzhou) in 2008 following BGCI’s collaboration in the development of China’s Strategy for Plant Conservation (CSPC) [157]. The CSPC highlighted China’s botanical wealth and the urgent need for conservation action. BGCI activities focus on collaborative action with botanic gardens and other botanical institutions in China to develop and implement practical conservation initiatives for the country’s threatened native flora to address the targets of the CSPC. In the past 13 years, through the Global Trees Campaign (a partnership between BGCI and Fauna and Flora International) BGCI has funded the protection and restoration of more than 70 threatened trees in China. 

A summary of the work BGCI has been implementing with Chinese partners over the past 10 years was recently published with more than 20 case-studies on the integrated conservation of rare and threatened woody plants [158]. One such example is *Bretschneidera sinensis* an endangered tree native to China. Through comprehensive field surveys, wild populations were identified, and seed collected for storage in the South China Botanical Garden seed bank. Additional seed was collected for reintroduction activities including the reintroduction of 1000 seedlings in Dongguan Forest Park and Shimen National Forest Park of Guangzhou city and 300 seedlings into Nankunshan Mt. Nature Reserve.

The **Global Tree Seed Bank Project** (GTSBP) is one of Kew’s major science-based plant conservation programmes. Funded by the Garfield Weston Foundation it aims to secure (in safe, long-term storage) seeds of at least 3500 tree species from across the world. The Latin America programme of the GTSBP was initiated in 2015 and delivered through two projects focused on useful trees in **Mexico** and the **Dominican Republic**. 

Mexico is in the top five countries in terms of floral diversity (species richness), and more than 50% of the plant species are endemic [159]. There are ca. 3000 native tree species that have been characterised for their uses, distribution, conservation status and endemism [160]. However, more than 30% of the tree species are threatened due to deforestation and/or climate change. The Science-Based Conservation of Tree Species in Mexico Project addressed this by implementing an integrated conservation programme for endemic, protected and useful tree species, important for the livelihoods of rural communities. Work was undertaken in collaboration with the Facultad de Estudios Superiores Iztacala de Universidad Nacional Autónoma de México (FESI-UNAM). Around 400 species have been conserved ex situ, in country and as duplicates at the MSB, and technical and scientific information has been produced to support the national reforestation programme led by the National Forestry Commission (CONAFOR), including seed germination and propagation requirements. A key challenge is promoting the use of more native species to diversify local nurseries which rely on the economic benefits obtained by selling and distributing seedlings. A lack of information about the uses and propagation of native trees other than timber species (mostly *Pinus*) means demand is very low. To overcome this challenge, in collaboration with partners (University and NGOs) species profile sheets (in the local language) were produced and distributed, describing their germination and propagation requirements, uses, phenology, distribution and conservation status to promote their utilisation in reforestation programmes.

In the Caribbean region, the vegetation cover in the island of Hispaniola, is under threat from the expansion of agriculture and development for tourism, and the unsustainable logging for charcoal production. Kew has worked with the Jardín Botánico Nacional (JBN) “Dr. Ma. Moscoso” of Santo Domingo through the MSBP since 2007 under an Access and Benefit-Sharing Agreement with the aim to support the conservation and sustainable use of the Caribbean native flora. Through this collaboration, a new seed bank was established in 2017 [161] together with a conservation programme, which included the provision of technical and scientific support, capacity building and seed research for ex situ and in situ conservation. The Hispaniola Island climate is mainly tropical, and as in most tropical regions, the percentage of species with recalcitrant (non-bankable) seeds is higher than in temperate climates. Thus, one of the challenges faced was the identification or prediction of native species whose seeds could not be conserved under traditional seed banking conditions [162]. The joint Garfield Weston funded project Saving threatened forests of Hispaniola focused on protecting the forests on the island, by researching, conserving and propagating the seeds of native useful tree species and supporting reforestation activities. Seeds of 250 tree species have been banked and planting activities have been carried out in degraded areas, seedlings have been donated for the establishment of a new Botanic Garden in Santiago and used for enhancing the vegetation of urban parks and other green spaces in Santo Domingo. The collaboration has helped the JBN to become a key player for the Ministry of Environment and Natural Resources in the Dominican Republic in seed conservation and the provision of plant material and information for restoring and recovering protected as well as urban and semi urban areas.

Under the umbrella of the **Great Green Wall Initiative**, Kew’s pilot project (2013–2020) built a restoration model and generated environmental and socio-economic information to support larger-scale restoration projects in similar contexts and conditions in the Sahara and Sahel regions [163]. Under Access and Benefit-Sharing Agreements, this collaborative project was implemented in partnership with national institutions and local communities in the cross-border zone between three countries: Bankass in Mali; Djibo and Dori in Burkina Faso; and Téra in Niger. A participatory approach was used to select native useful plant species adapted to local conditions, and those that are important to the communities’ livelihoods [164]. The most environmentally well-adapted and economically relevant species were prioritised and authenticated, and seeds of 84 useful woody and herbaceous species were collected from the wild and stored according to international standards in national seed banks with duplicates at the MSB. Seed accessions were tested and research on seed biology and ecology was carried out at Kew to support the conservation and propagation of species. In collaboration with local communities, seeds of 55 woody and herbaceous species were propagated and planted to restore 2235 ha of degraded land and create sustainable income-generating opportunities for up to 32,000 people. Over 1,000,000 seedlings of the selected species were planted and monitored in around 200 experimental plots [164]. An assisted natural regeneration approach was used for some of the most important species, such as *Guiera senegalensis*, as an alternative to reforestation [165], while the planting of tall bare roots of *Adansonia digitata* (Baobab) was found to have a better chance to resist and survive harsh climatic conditions and the species is highly valued by farmers [166]. Over 100 village technicians have been trained in nursery management, tree planting, forest restoration and establishment of demonstration plots during the duration of the project. The project has generated capacity at national and regional level to develop, plan and implement science-based restoration programmes aimed at reestablishing the natural capital of the vegetation and supplying the resource base for the enhancement of local livelihoods.

## 5. Future Directions

In summary, both living and seed collections held in botanic gardens and seed banks offer huge long-term opportunities for the conservation of wild plant diversity. Such collections are a key input into agricultural research and the development of improved crop varieties, with plant breeding depending fundamentally on the availability and accessibility of useful genetic resources, such as those found in crop wild relatives. In addition, WEP can provide the basis of new crops. Some examples of the value to humanity of successful long-term maintenance of a wide spectrum of genetic diversity are provided by Bretting [167], and other uses of seed and plant collections are described in this paper. 

Beyond their immediate use value, seeds stored in genebanks represent a form of insurance against the loss of genetic material in situ. Furthermore, and in common with other scientific collections, they may well have uses beyond their original purpose. The use of herbarium specimens to investigate the impacts of climate change is well recognised, and it may be that seeds stored for the long term will offer opportunities for future research, answering questions so far not asked and using tools not yet developed. 

There are, however, challenges to be addressed. There are gaps in the geographic, taxonomic and genetic coverage of collections, as well as gaps in coverage of useful plants. It is apparent that ex situ collections and conservation expertise continue to sit outside the countries with the greatest plant diversity. It is, however, encouraging to see that this situation is changing, through investment in people and facilities within biodiverse regions, but more needs to be done.

Availability of funding on a scale commensurate with the biodiversity crisis remains an issue. The conservation of plant diversity needs a step change in scale and investment if we are to prevent the predicted extinction of two in five plants, and one in three trees. The shortage of funding means that prioritisation of which plants are conserved must take place—and this can take many forms depending on the organisation undertaking the conservation initiative. For example, botanic gardens and seed banks have long focused on the most threatened species, including endemics, but also on economically important species, including medicinal plants and CWR. This does not, however, mean that those plants not prioritised have no intrinsic value of their own, and often reflects limitations in our knowledge of plant properties, uses and threat status. 

The costs associated with long-term storage pose another constraint and mean that choices need to be made on what to conserve and how to do this, and there is limited information available to guide such choices. Detailed knowledge of local, regional, and global patterns of plant diversity are often lacking [168], and as mentioned, seed banks are often not located where the greatest diversity exists. Savings should not, however, be made through identifying and removing duplicates from collections, as holding multiple accessions from the same population through time and from different populations of the same species are of great conservation value. False savings should also be avoided, for example, devaluing material that is used the least. At the same time, duplication between multiple types of ex situ conservation should be monitored and could provide potential for rationalising collections. 

How long material is held is a further consideration. While funds are generally used for the acquisition of plant genetic resources, their long-term maintenance and monitoring also needs to be funded, often directly by the institutions holding the collections. The MSB was built to last 500 years, and most species banked will survive 10 s to 100 s of years under storage conditions, but it is questionable whether sufficient funds will be available to support the long-term ex situ conservation of the world’s genetic resources [168]. 

The increasing use of genomic information in breeding may suggest a move towards dematerialisation of collections and a greater focus on storing genomic data only. We would of course argue that there are many more uses of seeds beyond their use in breeding programmes and the ability of scientists to work usefully with DNA alone is still some way in the future. We therefore do not believe that conserving dematerialised DNA will be a useful plant conservation strategy for some time to come. Another challenge is the increasing complexity of issues around the ownership and control of genetic resources as well as the sensitivity of the legal and political situations. 

Quality control must continue to be addressed, and improved monitoring systems put in place. While a number of standards and guidelines have been published to guide seed banking within the botanic garden sector, it is not clear to what extent these standards are being followed and how much of the seed held by botanic gardens and other institutes is in fact viable and usable. Within the European plant genetic resources community, a seed bank quality system has been put in place which provides a set of policies, processes and procedures that are to be followed by all members of the European Genebank Integrated System (AEGIS) to assure an appropriate quality of activities. The system requires all members to develop an operational genebank manual based on a common template that documents the operating procedures and standards of the genebank. In 2019, in the framework of the EU-funded project GenRes Bridge, three European genebanks were evaluated as part of a pilot project. The project allowed the genebanks to review each other on the basis of their Genebank Manuals and provided a mentoring system to help the genebanks address quality issues identified during the reviews [169]. This pilot project may provide valuable lessons to guide efforts to improve the quality of botanic garden seed banks. 

Two further areas stand out as opportunities to improve ex situ conservation for the future: technological advances and networks. Technological advances in cryobiotechnology are already helping some seed banks conserve existing collections of wild species whose seeds are short-lived under conventional seed banking conditions, as well as species with intermediate or recalcitrant seeds (Figure 1). The process for the latter is, however, research intensive as species require tailored protocols to be developed for them to optimise conservation outcomes. The flora of highly diverse and threatened regions are a priority for conservation, and these tend to fall in the tropics—which have both a geographical gap in existing collections, and a technological gap in relation to the conservation of ‘exceptional species’, which are more prevalent in these regions. There is a need to increase the availability of cryopreservation options for these species through provision of facilities, or access to existing facilities currently used for different purposes (e.g., crops), and through training. Developing and strengthening networks within tropical regions will greatly help. 

We have highlighted the importance of networks for sharing knowledge, data, expertise and access to facilities as well as to raise the profile of plant conservation and leverage funding. It is important to grow and maintain conservation networks, not just within the botanic garden and seed bank sectors, but by incorporating other practitioners and local communities working towards the conservation of a given species, habitat or flora. Such networks can help establish integrated conservation planning from the outset of programmes, and lead to greater impacts for conservation. In addition, greater linkages need to be formed between the conservation and agricultural worlds to ensure that we hold the material needed to create resilient crops of the future and to diversify the food plants grown as crops. Forming networks that interlink these sectors will help to increase the impact of global conservation efforts, avoid unnecessary duplication and prioritise resource allocation, as was largely achieved through the Adapting Agriculture to Climate Change project. The diversification of metacollections, currently focused on ‘exceptional species’ to a wider array of threatened plant taxa should also be encouraged as this networking approach greatly enhances the value of individual collections and improves conservation outcomes for the species involved. 

Botanic gardens and seed banks are well placed to respond to the biodiversity crises, but their conservation efforts need to be massively scaled up and supported by long-term funding. They also need to be coordinated across institutions, sectors (government agencies, universities, NGOs, etc.), geographies, and political boundaries [34]. Technological advances offer hope for the ex situ conservation of all plants—we now need to act on this possibility. 

## Data Availability

Data sharing not applicable.
